# Membrane Bioreactor Technology: The Effect of Membrane Filtration on Biogas Potential of the Excess Sludge

**DOI:** 10.3390/membranes10120397

**Published:** 2020-12-06

**Authors:** Magdalena Zielińska, Katarzyna Bernat, Wioleta Mikucka

**Affiliations:** Department of Environmental Biotechnology, University of Warmia and Mazury in Olsztyn, Słoneczna St. 45G, 10-709 Olsztyn, Poland; magdalena.zielinska@uwm.edu.pl (M.Z.); bernat@uwm.edu.pl (K.B.)

**Keywords:** MBR, ultrafiltration, microfiltration, membrane fouling, municipal wastewater, biogas production

## Abstract

Although the membrane bioreactor technology is gaining increasing interest because of high efficiency of wastewater treatment and reuse, data on the anaerobic transformations of retentate are scarce and divergent. The effects of transmembrane pressure (TMP) in microfiltration (MF) and ultrafiltration (UF) on the pollutant rejection, susceptibility of ceramic membrane to fouling, hydraulic parameters of membrane module, and biogas productivity of retentate were determined. Irrespective of the membrane *cut-off* and TMP (0.2–0.4 MPa), 97.4 ± 0.7% of COD (chemical oxygen demand), 89.0 ± 4.1% of total nitrogen, and 61.4 ± 0.5% of total phosphorus were removed from municipal wastewater and the permeates can be reused for irrigation. Despite smaller pore diameter, UF membrane was more hydraulically efficient. MF membrane had 1.4–4.6 times higher filtration resistances than UF membrane. In MF and UF, an increase in TMP resulted in an increase in permeate flux. Despite complete retention of suspended solids, strong shearing forces in the membrane installation changed the kinetics of biogas production from retentate in comparison to the kinetics obtained when excess sludge from a secondary clarifier was anaerobically processed. MF retentates had 1.15 to 1.28 times lower cumulative biogas production than the excess sludge. Processing of MF and UF retentates resulted in about 60% elongation of period in which 90% of the cumulative biogas production was achieved.

## 1. Introduction

To achieve high-quality effluents in the municipal wastewater treatment plants (WWTPs), membrane bioreactors (MBRs) are increasingly employed [1,2]. Although conventional activated sludge systems are still cheaper to use than MBRs, the costs of membranes and MBR operation are decreasing and MBRs offer greater opportunities for water reuse as the most rapidly developing solution in wastewater treatment [3]. The MBRs integrate an activated sludge process with a permselective membrane that separates sludge flocs from the effluent. Thus, the technology allows avoiding sludge washout from the system at short hydraulic retention time [4]. Microfiltration (MF) and ultrafiltration (UF) membranes that replace secondary clarifiers in the MBRs are complete barriers for the flocs, which allows maintaining a high biomass concentration in the bioreactor, up to 30 g/L. As a result, volumetric reaction rates that are about twice higher than those in conventional activated sludge systems allow for small footprint [5]. Effective biomass retention leads to a very long sludge age and low sludge production [3]. At high biomass concentration, long solids retention time (SRT), and low food-to-microorganisms ratio (F/M), bacteria primarily utilize the energy supply for their maintenance metabolism [6]. Therefore, they can mineralize substrates rapidly, resulting in obtaining above 90% of organic matter removal from wastewater [7]. In a full scale A2O-MBR, concentrations of organic compounds (COD), NH_4_^+^-N and total phosphorus (TP) removal efficiency was more than 90.0, 98.2, and 96.2%, respectively [8]. In a membrane system connected with aerobic granular sludge, 94.84% of total nitrogen (TN) and 97.07% of TP was removed [7]. In an MBR combined with post-denitrification, the removal of TN and TP was about 85% and 87%, respectively [9]. Post-denitrification was added because the effective removal of phosphorus and nitrates is deteriorated in some facilities because of the fact that huge aeration rate for membrane cleaning results in high concentration of dissolved oxygen in the mixing liquor recycled from a membrane tank to an anaerobic or anoxic tank.

The most commonly used submerged MBRs usually require less energy to run but need a larger membrane surface area than external cross-flow MBRs [1]. This results from higher susceptibility of submerged membranes, which are usually polymer membranes, to fouling. Fouling is the main obstacle for optimal membrane functioning, particularly in MBRs which are operated under the conditions of high biomass concentration. Suspended particles (sludge flocs, microorganisms, and cell debris), colloids, and solutes are the main reasons for fouling in MBRs [10]. Ceramic membranes are less prone to fouling and provide increased concentration polarization control than polymer ones, because the bonding between membranes and foulants is weaker because of more hydrophilic surface of ceramic membranes [11]. They are particularly suitable when the feed contains a relatively high proportion of large particles. Ceramic membranes, due to high variety of materials used for their fabrication, are highly resistant to corrosive chemicals, have favorable mechanical strength and long lifespan, stability at high temperatures (they may be sterilized with steam), possibility of dry storage, good antimicrobial ability, and high separation efficiency [12]. They are rarely used in full-scale facilities, mainly because of the cost. However, despite the lower costs of polymeric membranes, ceramic membranes may be competitive with polymeric membranes over the long term. In addition, there are reports in the literature on the development of new methods of production of low-cost ceramic membranes based on locally available raw materials such as clay, kaolin, diatomaceous earth, etc., [13]. These low-cost membranes have water permeability values similar to those of conventional ceramic membranes. All these characteristics of ceramic membranes indicate that they should be investigated to develop their use in large-scale environmental applications.

Although wastewater treatment in MBRs is well recognized, there is no data on the processing of the excess sludge (retentate) and, particularly, on the susceptibility of the retentate to biological stabilization. First, MBRs were mainly applied in small WWTPs. Now, MBRs are also operated in large facilities [14], in which anaerobic stabilization of sludge is profitable. Data are available only on the use of anaerobic MBRs, in which biogas produced by converting up to 98% of COD [15] had 80% or even 90% of CH_4_ [16] and the electricity generated covered the MBR energy demand, which varies from 0.5–0.7 kWh/m^3^ [17] to 2.0–12.0 kWh/m^3^ [18]. In aerobic MBRs, despite the effective sludge thickening, anaerobic processing of excess sludge is sometimes questionable. Theoretically, because of low organic loading (F/M of about 0.1 kg COD/(kg·d)), the production of excess sludge in the MBRs is very low (<0.1 kg mixed liquor volatile suspended solids (MLVSS)/kg COD) resulting in low MLVSS/MLSS (mixed liquor suspended solids) ratio of about 50% [19]. Therefore, some authors indicated that the introduction of an anaerobic digestion stage may not be useful because of the low production of biogas (0.07–0.18 m^3^/kg of volatile solids added) [20]. However, in practice, the sludge yield obtained at SRT of 45 d and F/M of 0.02 kg COD/(kg MLVSS·d) was similar to that obtained at three times shorter SRT and three times higher F/M [20]. In the full-scale WWTP operated in the MBR technology, the MLVSS/MLSS ratio was 73% [21], which was like that in conventional activated sludge process. Thus, anaerobic digestion of the excess sludge from the MBR may be considered in these WWTPs in which it is profitable.

In sewage sludge processing, various pretreatment technologies have been developed to improve the anaerobic digestion by accelerating the hydrolysis of sludge. As one of pretreatment methods, high pressure results in sludge floc breakage and cell rupture and causes a release of organic substances which become easily accessible to subsequent biological degradation. At 200–400 bars, COD release due to sludge disintegration might relate to the release of extracellular polymeric substances (EPS) and cell components during cell lysis [22]. A pretreatment at 150 bars gave 23% sludge reduction and biogas production increased by 30% [23]. Similar results can be obtained using membrane installation in the MBR technology. For each m^3^ of permeate effluent, 20 to 30 m^3^ of bulk liquid must pass over the membrane; a velocity of 1–5 m/s and a pressure of 100 to 600 kPa must be used [24]. Because of cross flow conditions and high-speed circulation pumps, strong shearing forces occur in the installation, causing an intensive degradation of biomass particles. This should result in better availability of the organic substrate, present in the excess sludge, for anaerobic transformations [24].

On the other hand, floc degradation by high hydraulic shearing forces can reduce the specific metabolic activity and biogas production. In the membrane thickening of potato starch wastewater, a 50% activity loss was reported after 20 time circulating the sludge and a 90% loss after 100 time circulating [24]. This shearing stress may disrupt sludge flocs, alter cell viability, and cause a release of intracellular or extracellular compounds [24]. When circulating mixed liquor in the MBR, floc breakage, release of organics from cells, and deterioration of microbial activity, expressed by 78% reduction in specific oxygen uptake rate and thus lower substrate utilization rate, were reported [25].

Although MBR technology has been extensively developed and employed for wastewater treatment, to the best of our knowledge, data are lacking on the effect of membrane processes on the characteristics of retentate and its utilization for biogas production. In addition, the use of ceramic membranes, which offer advantages in terms of fouling resistance, has received little attention in the context of MBR operation. Therefore, the present study aims to improve knowledge of these aspects of MBR operation. The main objective of this study was to investigate the effect of membrane *cut-off* and TMP on the composition of the retentate and thus on its biogas production kinetics, with a focus on comparing that kinetics with the kinetics of biogas production with activated sludge collected from the secondary clarifier of a conventional WWTP. To gain insight into how replacing the secondary clarifier with a membrane module would influence WWTP performance, the effects of membrane *cut-off* and TMP on the efficiency of removal of TSS, COD, BOD (biochemical oxygen demand), TN, and TP were also investigated. A novel aspect of this study is the use of ceramic membranes in a side-stream configuration for activated sludge separation, including the determination of the effects of *cut-off* and TMP on the susceptibility of these membranes to fouling, as reflected by changes in permeate flux and membrane resistance. The results extend current knowledge on the use of ceramic membranes for separating activated sludge from treated wastewater in municipal WWTPs.

## 2. Materials and Methods

### 2.1. Characteristics of WWTP

The feed for membrane filtration, which was a mixture of treated wastewater and activated sludge flowing from biological reactors to the secondary clarifier, was collected from in a full-scale municipal WWTP located in northeast Poland. The WWTP had an average flow of 35,000 m^3^/d, a population equivalent of about 342,000, and the pollutant indicators in the influent of 960 ± 44 mg COD/L, 510 ± 24 mg BOD/L, 410 ± 22 mg TSS/L, 92 ± 5 mg TN/L, 11 ± 2 mg TP/L. The mechanical part of the WWTP embraces grates, grit chambers, and primary clarifiers, whereas the biological part includes a dephosphatation chamber, circulating reactors with automatically adjusted surface aeration to provide anoxic/aerobic conditions, and secondary clarifiers. The effluent is discharged into the river. Primary sewage sludge is thickened, and volatile fatty acids are produced to support the biological dephosphatation. The mixture of primary sludge and secondary sludge is anaerobically digested to produce biogas, then dewatered, and finally thermally processed. The feed for membrane filtration was characterized by 4500 ± 212 mg MLSS/L, with 73% of MLVSS. 

### 2.2. Membrane Filtration

The experiment on separation of activated sludge from the effluent was conducted in the installation that consisted of a 10-L process tank, a CRN(E) high-pressure pump (Grundfos), a heat exchanger, a prefilter (1 mm mesh), a flowmeter, a membrane module located outside the process tank, pressure gauges at the feed and retentate sides of the membrane module, a restricting valve to pressurize the feed to the desired value, a line to circulate the retentate to the process tank, and a line to discharge permeate from the installation. The pressure-driven membrane module contained a ceramic asymmetric membrane (Inside CéRAM, Tami Industries, Hermsdorf, Germany), which was a kind of multi-tube membrane because of the presence of 23 channels inside. The membrane was fabricated from TiO_2_ and ZrO_2_ and characterized by a length of 300 mm, an external diameter of 25 mm, a hydraulic diameter of each channel of 3.5 mm, a filtration area (A) of 0.1 m^2^, a specific area of 680 m^2^/m^3^, and chemical resistance in the range of 0–14 pH. The membrane permeability for deionized water was about 1500 L/(m^2^∙h·bar) for MF and 500 L/(m^2^∙h·bar) for UF. 

Six experimental series with the use of MF membrane (pore size of 0.45 µm) and UF membrane (*cut-off* of 150 kDa) were conducted, in which constant TMPs of 0.2, 0.3, and 0.4 MPa were used (Table 1). For each experimental series, 10–18 L of feed was introduced into the process tank. The installation was operated in a cross-flow system with an initial feed flow velocity of 17–22 L/min at 21 ± 1 °C. During the filtration cycle, permeate was yielded from the membrane system, whereas the retentate was circulated back to the process tank. Thus, the flow velocity was the velocity of both the feed and the retentate that circulated in the loop, and the suspension circulating in the installation became more and more concentrated as the experiment proceeded. Because of the lack of automatic backwashing, the membrane installation was operated in a batch mode. Therefore, each experimental series relied on conducting a permeation test. In these tests, permeate flux was monitored by measuring the time (t) necessary to obtain given volume of permeate to the moment of obtaining 50% permeate recovery (volumetric concentration factor of 2). The installation was initially run to eliminate about 2 L of distilled water that was present as dead volume in the system. Each of the filtration series was conducted in duplicate; the arithmetic mean of two measurements for each series is presented.

After each permeation test, the installation was washed according to producer’s recommendations, in the following sequence: with deionized water, with 2% NaOH solution, with deionized water to obtain neutral pH, with 1% HNO_3_ solution, and finally with deionized water to obtain neutral pH. The temperature of the washing fluids was 21 ± 1 °C. Washing was considered complete when 95–97% of the initial permeation flux of deionized water was recovered.

### 2.3. Analytical Methods

In feed, permeates and retentates, the concentrations of organic compounds (COD), TP, TN, NO_2_-N, and NO_3_-N were measured spectrophotometrically with cuvette tests (Hach Lange GmbH, Duesseldorf, Germany). BOD_5_ was measured with a DIN EN 1899-1/EN 1899-2 EPA method using OxiTop WTW Wissenschaftlich-Technische Werksträtten GmbH. The concentrations of NH_4_-N, MLSS, MLVSS and the settling capacity of activated were measured according to Hermanowicz et al. [26]. These analyses were also carried out in samples of raw and treated effluent from the WWTP, from which the activated sludge was collected. The morphology and size of activated sludge flocs in the feed to membrane filtration and in retentates were evaluated with the use of Nikon Eclipse microscope combined with Lucia 4.82 software (Laboratory Imaging, Prague, Czech Republic).

Biogas production from the excess sludge taken from the secondary clarifier of the full-scale WWTP (sewage sludge (SS)), and the production from MF and UF retentates were each determined in triplicate (for each sample) in a respirometric OxiTop system (500 mL bioreactors with individual heads). The gas production tests (GP) lasted 21 days (GP21) and were carried out in batch assays [27]. Biogas production, resulting from decomposition of organic matter, causes an increase in pressure in the gas head-space above the liquid, which is measured by the manometer inside the head. The pressure values were stored in the memory of the recording device and then used to calculate the volume of the biogas, employing the ideal gas law. Finally, the biogas production was expressed as L of biogas per mass of COD added (L/kg COD). The temperature of the measurement was 37 ± 1 °C, and bioreactors were kept in a thermostatic incubator. The inoculum (ca. 100 mL) was added to each bioreactor along with an appropriate dosage of the tested sample. As the inoculum, fermented sludge from the closed mesophilic fermentation chambers operated in the local WWTP was used. The characteristics of the inoculum were pH ca. 7.0, total solids (TS) ca. 2%, volatile solids (VS) ca. 70% of TS. The samples of SS and the MF and UF retentates were analyzed for the COD concentration of the whole sample (liquid and solid mixture). Based on COD concentrations, the dosage of the sample was calculated to assure the starting load of 5 kg COD/m^3^, which is close to the organic loading rate used in digesters operated under technical scale (5 kg COD/(m^3^∙d)). The biogas production from the inoculum alone was subtracted from the total biogas production of the sludge and inoculum combined. This allowed determination of the biogas production of the investigated sludge samples. Before measurement, after filling with the inoculum and the sludge sample, each bioreactor was flushed with N_2_. The lateral connections of the bottles were sealed with rubber stoppers. The contents of the bioreactors were mixed. After 21-day biogas production, overall biogas composition was measured. GA200+ automatic analyzer (Geotechnical Instruments, Coventry, Great Britain), which measured the percentage of methane and carbon dioxide in the biogas, was used to determine the biogas composition in the head space of the OxiTop bioreactors.

### 2.4. Calculation Methods

The total efficiency of pollutant removal was calculated based on concentrations in the influent to the analyzed WWTP and in the permeates. The concentrations of pollutants in feed (*C_F_*), permeates (*C_P_*) and retentates (*C_R_*), the volumes of feed (*V_F_*), permeates (*V_P_*) and retentates (*V_R_*) obtained under each operational conditions and deionized water flux (*J_w_*) were used to calculate the basic parameters of the membrane, such as the retention coefficient (*R*), permeate flux (*J_V_*), normalized flux (α), permeate recovery (*Y*), volumetric concentration factor (VCF), and total hydraulic resistance of the membrane (*R_t_*) (Equations (1)–(8)).

(1) $$R=\left(1-\frac{C_{P}}{C_{F}}\right)\cdot 100\%\;(\%)$$

(2) $$J_{V}=\frac{V_{P}}{t\cdot A}\;(L/(m^{2}\cdot h))$$

(3) $$\alpha =\frac{J_{V}}{J_{W}}\;(–)$$

(4) $$Y=\frac{V_{P}}{V_{F}}\cdot 100\;(\%)$$

(5) $$VCF=\frac{V_{F}}{V_{R}}\;(–)$$

(6) $$R_{t}=\;\frac{\mathrm{TMP}}{J_{V}\cdot µ}\;(m^{-1})$$

*R_t_* = *R_m_* + *R_f_* (7)

where: *µ* (Pa·s) was the solvent dynamic viscosity; *R_m_* (m^−1^) was the intrinsic membrane resistance calculated for water flux through pristine membrane; *R_f_* (m^−1^) was the fouling resistance as the sum of the resistance deriving from cake layer (*R_c_*) and from the clogged pores (*R_p_*) [28].

(8) $$R_{m}=\;\frac{\mathrm{TMP}}{J_{w}\cdot µ}(m^{-1})$$

*R_p_* was measured by filtering deionized water after flushing the membrane surface to wash out the cake layer. *R_c_* was calculated by subtraction of *R_p_* from *R_f_*.

To estimate the adsorption of pollutants (COD and TSS) on the membranes, the mass balance approach was employed. The mass balance assumes that mass of the pollutant in the feed (*C_F_V_F_*) is the sum of the mass in the permeate (*C_P_V_P_*) and in the retentate (*C_R_V_R_*). First, the mass of pollutants in the retentates in one filtration cycle was calculated by subtracting the mass in the permeate from the mass in the feed. Then, the actual mass of pollutants was measured in the analytical assessment of the retentates. The difference between the calculated and actual mass of COD and TSS in the retentates was considered as the mass that was adsorbed on the membrane.

In addition, based on the actual masses in feeds, permeates, and retentates, the adsorption of COD/TSS on the membranes was expressed as the percentage value (*Ads*, Equation (9)):

(9) $$Ads=\left(1-\frac{C_{R}V_{R}+C_{P}V_{P}}{C_{F}V_{F}}\right)\cdot 100\%\;(\%)$$

Biogas production followed pseudo 1st order kinetic:

(10) $$C_{t}=C_{0}\cdot \left(1-e^{-k\cdot t}\right)$$

where: *C_t_* (L/kg COD) was the cumulative biogas produced at digestion time *t* (days); *C*_0_ (L/kg COD) was the maximal biogas produced; *k* (d^−1^) was the kinetic coefficient of biogas production. The *C*_0_ and *k* were obtained by non-linear regression analysis with Statistica 13.0 (StatSoft). *R*^2^ coefficient was used to evaluate the fitting the model to experimental data. To conduct the statistical analysis of the results, after checking the hypothesis about the normality of variable distribution using the Shapiro–Wilk test, the significance of differences between the results was verified with the ANOVA and Tukey-HSD test. A value of *p* ≤ 0.05 was defined as significant. The strength of the relationships between groups of results was determined using Pearson’s correlation coefficient (r).

The energy produced from the feedstock (*E*) was calculated as follows:

(11) $$E=P_{m}\cdot C_{m}\cdot \mathrm{VSS}\;(\mathrm{kWh}/m^{3})$$

where: *P_m_* (m^3^ CH_4_/kg *VSS*) was methane production, *C_m_* (9.17 kWh/m^3^ CH_4_) was the calorific value of methane, *VSS* (kg VSS/m^3^) was the *VSS* concentration in the feedstock to methane fermentation. 

## 3. Results and Discussion

### 3.1. Efficiency of Wastewater Treatment with the Use of Membrane Separation

The full-scale municipal WWTP, from which a mixture of wastewater and activated sludge was collected and fed into the membrane installation, was characterized by high efficiency of pollutant removal. However, very low settling ability of activated sludge (expressed by sludge volume index of 210 mL/g) resulted in flotation of sludge in the secondary clarifier and required very long retention time. For such a case, the replacing secondary clarifier with a membrane unit would be an advantageous solution because in the MBR technology the sludge settling ability does not influence the final quality of the effluent. 

**Figure 1:**
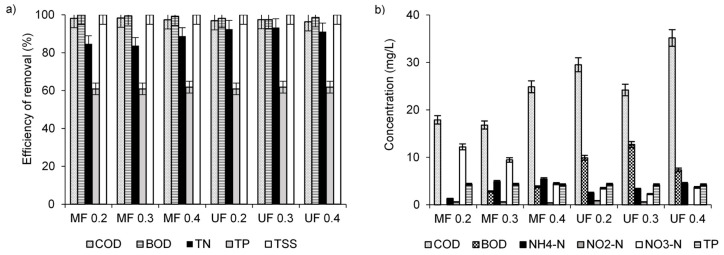
Efficiency of pollutant removal (**a**) and pollutant concentrations in the permeates (**b**) obtained in microfiltration (MF) and ultrafiltration (UF).

To test the possibility of replacing secondary clarifier with a membrane unit, the MF and UF modules with ceramic membranes were used to separate the activated sludge from treated wastewater. In such a system, TSS were completely rejected from the feed (Figure 1a). In MF and UF, size exclusion is the major mechanism of rejection. However, despite the bigger pore sizes in the MF membrane, total efficiency of COD removal was about 97.4 ± 0.7%, irrespectively of the membrane *cut-off* and of the TMP. This similar rejection of COD in MF and UF could have resulted from two reasons. First, the membrane fouling decreased the effective diameters of the pores, which allowed for the retention of molecules that were smaller than would be expected based on the nominal pore sizes [29]. Second, mainly suspended particles larger than 0.45 µm, which were efficiently rejected in MF, were responsible for COD in the feed. These hypotheses may be confirmed by BOD rejection. BOD removal efficiency was 99.6 ± 0.4% in MF and 98.0 ± 0.5% in UF. MF membrane may be more susceptible to fouling than UF membrane [30], which decreases the MF pores more intensively. However, the COD remaining in UF permeates may have resulted from the presence of low-molecular weight (below 150 kDa) organics such as proteins, saccharides or humic substances [31]. The removal of TN and TP in MF and UF is possible only when these compounds are present in organic form and are incorporated into the solid fraction after their assimilation by microbial cells or when these compounds are adsorbed on particles of organic matter. Thus, there were no differences in the removal of TN (89.0 ± 4.1%) and TP (61.4 ± 0.5%) in MF and UF. The increase in TMP accelerates water transport through the membrane and decreases the concentration of the pollutants in permeate [32]. On the other hand, when operating MF for treating real dairy wastewater at pressures from 207 to 414 kPa, an increase in pressure resulted in an increase in COD in the permeate due to an enhanced convective flux through the membrane [33]. In the present study, the TMP in the range 0.2–0.4 MPa did not influence the efficiency of pollutant removal. All these results indicate that the effect of increasing pressure on the efficiency of pollutant removal depends on the specific conditions in a membrane module, and most likely depends on whether the module is operated below the critical flux [33].

Comparing pollutant loads in the effluent from the analyzed WWTP working with a secondary clarifier with pollutant loads obtained when ceramic membranes were employed for the separation of sludge, the use of membranes reduced approximately three times (data not shown) COD and BOD loads in the final effluent. TN and TP loads in permeates were only slightly smaller than in the outflow from the secondary clarifier. This indicated that total removal of TN and TP mainly depended on the biological treatment. Therefore, the optimization of operational conditions to improve denitrification, biological accumulation of phosphate, or the introduction of chemical precipitation of phosphate would be necessary to increase the efficiency of nutrient removal. 

The analyzed WWTP operated in a conventional manner produces effluent that meets the criteria for wastewater discharge into an aquatic environment. At present, because of the lowered quality and availability of water resources, wastewater reuse has gained interest as an environmentally and economically viable option for municipal activities. The US Environmental Protection Agency [34] suggests guidelines for reuse of water from municipal wastewater treatment plants with a low contribution of industrial wastewater. The most promising solution for reuse is membrane separation, including MF and UF as more economical than e.g., nanofiltration because of their high flux with a low energy input [35]. The MBR effluent may be considered adequate for many reuse applications. In the present study, the use of ceramic membranes allowed obtaining a final effluent with very low pollutant concentrations (Figure 1b). Irrespectively of membrane *cut-off* and TMP, these concentrations did not exceed 35.2 mg/L (COD), 12.7 mg/L (BOD), 15.0 mg/L (TN, 63% in the form of nitrates), 4.3 mg/L (TP); the TSS concentrations were below the detection limit. Therefore, these permeates show promise for reuse, for example, in irrigation, because of TSS < 10 mg/L, COD < 100 mg/L, conductivity 250–750 mg/L, total dissolved solids < 450 mg/L, NO_3_-N 5–30 mg/L, and pH 6.5–8.3 [36]. When water of higher purity should be produced, the investigated system may be applied upstream of nanofiltration or reverse osmosis [18].

### 3.2. Hydraulic Capacity of the Membrane Installation

**Figure 2:**
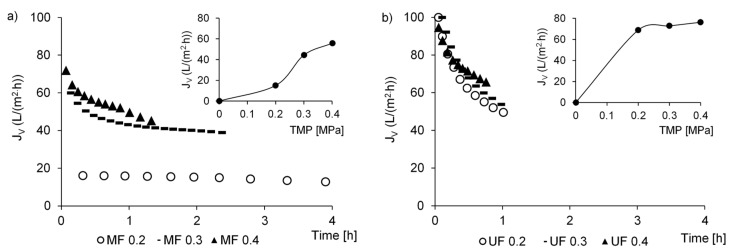
Changes in permeate flux in MF (**a**) and UF (**b**).

Based on permeation tests, the changes in J_V_ over time and the average J_V_ values were assessed (Table 2). In MF (Figure 2a), at TMP of 0.2 MPa, the initial J_V_ was very low (16 L/(m^2^·h)) and dropped to about 13 L/(m^2^·h) after almost 4 h when 50% of permeate recovery was obtained. An increase in pressure is one method of improving flux, therefore, the increase to 0.4 MPa resulted in an increase in the initial J_V_ to about 60 and 72 L/(m^2^·h) at 0.3 and 0.4 MPa, respectively. A gradual decrease in J_V_ was observed over time, because of progressive concentration polarization on the membrane surface and blocking of membrane pores by feed constituents [37]. Despite this flux deterioration, it took 2.3 and 1.3 h to obtain Y = 50%. In UF, the initial J_V_ was about 100 L/(m^2^·h) and it dropped sharply because of fouling and gave 50% permeate recovery after 1 h (0.2 MPa), 0.9 h (0.3 MPa), and 0.8 h (0.4 MPa) (Figure 2b). In MF, increasing pressures caused a linear increase in the average permeate flux (r = 0.96), indicating that, in the range of 0.2–0.4 MPa, there was hydrodynamic control of permeation flux that was driven by the pressure (Figure 2a (insert)). In UF, increasing pressures did not cause a linear increase in average permeate flux throughout the entire pressure range (Figure 2b (insert)). This may indicate that organic compounds were adsorbed on the membrane surface at TMPs of 0.3 and 0.4 MPa [33] and that the critical flux could have been reached at 0.3 MPa.

In UF, an increase in TMP from 0.2 to 0.4 MPa caused an increase in *R_t_* by about 90% (Table 2). This resulted from the fact that as the TMP increases, more solid pollutants accumulate on the membrane surface, and are compressed on the surface and form a gel layer, blocking the pores [28]. Despite this increase in *R_t_* and the fact that the pollutants present in the feed clogged the membrane pores at the beginning of the filtration process, which is seen from rapid decrease in *J_V_* with time, the assumed permeate recovery was obtained in substantially shorter time than in MF. This indicated that UF membrane was more hydraulically efficient, despite the smaller diameters of pores. It also resulted from the fact that MF membrane had higher filtration resistances than UF membranes. In MF, the values of *R_t_* were from 4.8 to 1.4 times higher than in UF at corresponding TMPs. Another indicator of membrane fouling, apart from a decrease in permeate flux, is normalized flux (α) as the ratio between permeate flux and deionized water flux. Independently of the membrane *cut-off* and the pressure, the values of α were much below 1, which indicates fast fouling (Table 2). Significantly lowest values of α in MF points on higher susceptibility to fouling than UF membranes under the conditions, in which a dense mixture of activated sludge and wastewater is the feed to the membrane installation. This is confirmed by Hwang et al. [38] who reported that membranes with larger pores were subjected to more severe fouling. The ratio between the size of the particles and size of the pores is more responsible for membrane fouling than only the size of the particles [39]. Particles of sizes similar or smaller than the pore diameter block the membrane pores, whereas particles larger than the membrane pores are retained on the membrane surface and washed out of the surface by flow forces. Also, Qu et al. [40], who investigated UF fouling caused by extracellular algal organic matter stated that membranes with larger pores exhibit higher reduction in flux but less adsorptive fouling. In the present study, such a case was observed at TMPs of 0.2–0.4 MPa. Despite lower intrinsic membrane resistance in MF (*R_m_* of 2.25·10^11^ m^−1^) than in UF (*R_m_* of 7.2·10^11^ m^−1^), in MF (larger pores), fouling was more intense than in UF (*R_t_* 4.8, 1.6 and 1.4 times higher, respectively) at simultaneous smaller percentage of adsorption of TSS (Ads-TSS) and COD (Ads-COD) in membrane pores at TMPs of 0.3–0.4 MPa (Table 2). However, both in MF and UF, reversible fouling (expressed as *R_c_*) dominated in the total membrane fouling (Table 2), which indicates that washing of the membrane allows recovering the permeate flux to a high extent. Based on the results of the present study and taking into account the principle that membrane systems should be run at the lowest possible TMP to be economically feasible, UF operated at the TMP of 0.2 MPa should be considered the optimal solution for the separation of the effluent from the activated sludge with the use of ceramic membranes.

**Figure 3:**
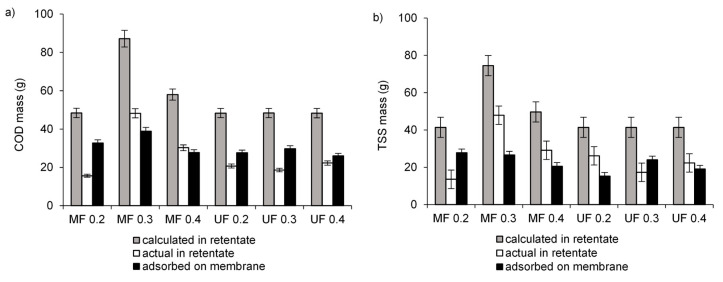
Calculated and actual mass of concentrations of organic compounds (COD) (**a**) and TSS (**b**) in retentates from MF and UF and mass adsorbed on the membranes.

The comparison of calculated mass of TSS and COD in the retentates with actual mass measured in the retentates revealed that some mass of TSS and COD was not present in the obtained retentates (Figure 3). This lower measured mass than the calculated mass indicates adsorption of pollutants in the membrane pores or on its surface. In Table 2, the percentage of adsorbed compounds was shown. There were no effects of TMP on the adsorption percentage in MF and UF. The highest percentage of TSS (67.2%) and COD (67.5%) adsorption was obtained in MF at the lowest TMP. It could have been the reason for the lowest permeate flux (Figure 2a). However, adsorption supported a simple size exclusion and allowed for similar efficiency of pollutant removal than that obtained in UF (Figure 1a).

### 3.3. Biogas Production

**Figure 4:**
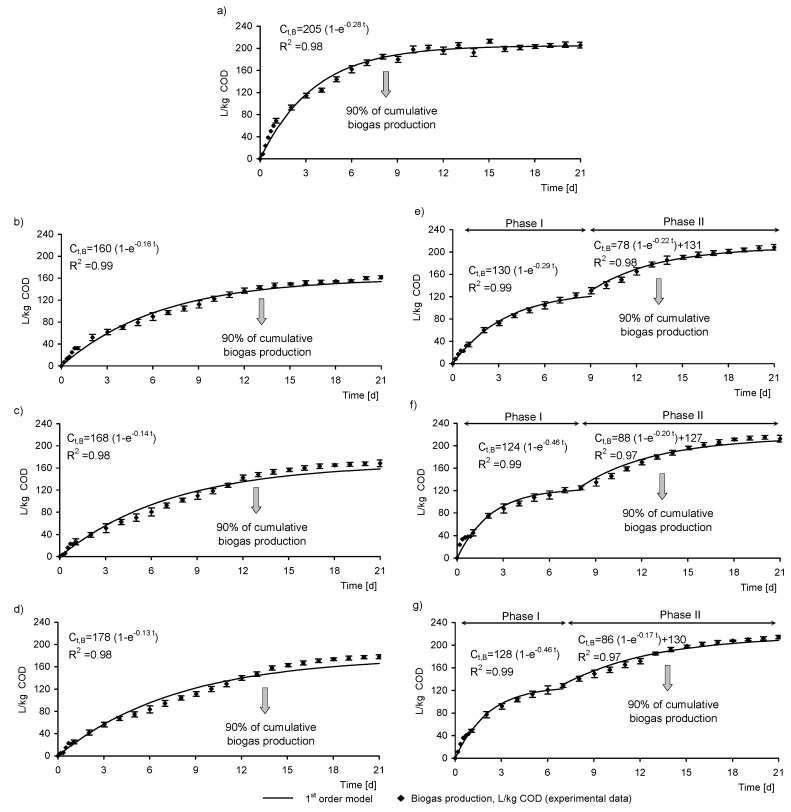
Biogas production from the SS and the retentates after UF and MF during GP21 test; (**a**) SS, (**b**) MF 0.2, (**c**) MF 0.3, (**d**) MF 0.4, (**e**) UF 0.2, (**f**) UF 0.3, (**g**) UF 0.4.

The SS from the analyzed WWTP and retentates from MF and UF were anaerobically treated to compare their biogas productivity. Figure 4 presents biogas production curves obtained during 21 days of anaerobic measurements with GP21 test. The cumulative biogas production from the SS was 205 ± 6 L/kg COD (Table 3). This cumulative biogas production after 21 days from UF retentates was from 208 ± 5 to 214 ± 3 L/kg COD and was similar to that from SS, and about 20% higher than that from MF retentates. This means that UF retentates may be more favorable for biogas production than MF retentates. 

The results of this study on the biogas production from SS and literature data showed that the biogas yield may change in a wide range and is expressed differently. For example, the biogas yield from the SS was 303 L/kg VSS [41], 234 L/kg VS (volatile solids) [42], or 188–214 L CH_4_/kg COD_fed_ [43]. Literature data show that the biogas yield from SS differ depending, for example, on the type of the wastewater treatment plant, type of the sludge (primary sludge, the excess sludge, granular sludge), and conditions in which the sludge was taken for analyses. The latter means that the biological sludge can be taken from the tank when a fresh portion of raw wastewater was supplied. Thus, the cells of the sludge may contain intracellular substances that may improve the biogas yield from the sludge. On the other hand, sludge from the secondary clarifier is suspended in treated wastewater deprived of nutrients, and sludge cells do not contain any storage materials. It may result in lower biogas production. The composition of the organic matter (e.g., carbohydrate, lipids, protein or fiber content) affects biogas production. Theoretically, a methane production from carbohydrates is 0.415 L CH_4_/g VS; from proteins, 0.496 L CH_4_/g VS; and from lipids, 1.014 L CH_4_/g VS [44]. Lignocellulosic biomass characterizes by low biodegradability and biogas production because of the complex structures of lignin and other cell-wall polysaccharides. Lignin is considered as not biodegradable in anaerobic environments [45]. The ratio of VS/TS in granular sludge from laboratory scale reactor treating municipal wastewater was 0.65 and hard-to-biodegrade lignin comprised ca. 54% of all the fiber materials [46]. These features of granular sludge caused the lowest biogas productivity (320–410 L/kg TS), and rate constants of biogas production (0.05–0.08 d^−1^), which are typical for substrates with high lignin content. Activated sludge taken from the wastewater treatment tank had a high content of proteins, and was rich in hemicellulose, constituting about 62% of all fibrous materials in the sludge. Its biogas production was very high, about 825 L/kg TS (1150 L/kg VS). Although there have been numerous studies concerning biogas production from SS, biogas production from retentates after MF or UF has not been investigated, which limits the amount of data available for discussion. 

In the present study, the pseudo-first-order kinetic model accounted for nearly all the variation in experimental data of biogas production of all investigated substrates (*R*^2^ > 0.97). Kinetic parameters from the model provide information about the biodegradability of the substrate. The substrate has higher biodegradability when the time with most intensive biogas production is shorter, and the values of the kinetic coefficient of biogas production (*k*) and the rate of biogas production (*r*) are higher. The period with most intensive biogas production is the time in which 90% of the cumulative biogas production is achieved (the grey arrow in Figure 4). Biogas production from SS and from MF retentates proceeded in one phase. The *k_I_* and *r_I_* from SS were highest (0.28 d^−1^, 57.1 L/(kg COD∙d)) from all one-phase biogas production curves. The period during which 90% of the cumulative biogas production was achieved for SS was shortest (8 days) among all tested substrates. MF retentates were characterized by 1.15–1.28-time lower biogas production and two-time lower values of *k_I_* and *r_I_* than SS. The *k_I_* decreased gradually when TMP increased. During anaerobic treatment of MF retentates, 90% of the cumulative biogas production was achieved after 13 days.

In contrast, the biogas production from UF retentates proceeded in two phases (Figure 4). In both phases, the experimental data were explained well by the first-order kinetic models. The *R*^2^ coefficients showed better fitting of the model to experimental data when the approach with two separate phases was used. After about 7–9 days of the measurement, biogas production achieved a first plateau. The duration of the phase I decreased from 9 to 7 days when TMP increased. After phase I, the cumulative biogas production constituted ca. 60% of the cumulative biogas production obtained after 21 days of anaerobic measurements (after both phases of biogas production). Then, biogas production started to increase, to finally achieve the second plateau. Particular components of organic substrates are biodegraded at different rates before they became a substrate for methanogens. Therefore, after 7–9 days of biogas production, a new portion of organics in UF retentates may become available and convert into biogas. Thus, the second phase of biogas production revealed. The *k_I_* for phase I increased from 0.29 d^−1^ for the retentates obtained at 0.2 MPa to 0.46 d^−1^ for the retentates obtained at 0.3 and 0.4 MPa. The *k_I_* affected the rate of biogas production from UF retentates; *r_I_* increased from ca. 37 L/(kg COD∙d) for retentate obtained at 0.2 MPa to ca. 58 L/(kg COD∙d) for retentates obtained at 0.3 and 0.4 MPa. The *k_II_* values for phase II were lower than those from phase I. Also, the *r_II_* was lower. In this phase, the *k_II_* and *r_II_* were lowest for the retentate after UF at 0.4 MPa. The fact that around 60% of the cumulative biogas production was obtained in the first phase of production means that remained biogas was produced in the second phase, for 12–14 days. Obtaining less biogas in the longer period slows down the kinetics of biogas production. The most available organic compounds are used rapidly when the digestion process starts. In the present study, at the beginning of the second phase of biogas production, a new portion of organics may have become available to the microorganisms for anaerobic decomposition. These organics were not as easily biodegradable as those in the first phase; therefore, the kinetic parameters of biogas production were lower in the second phase.

Although in both MF and UF retentates the period of most intensive biogas production was increased by about 60% as compared to that in SS processing, the kinetics of biogas production from MF retentate and from UF retentate differed. The rate of biogas production was lower from MF retentate and, in addition, two phases of biogas production were observed when processing UF retentate. Despite decreasing the effective diameter of pores in MF due to intensive fouling, the *cut-off* of the UF membrane was still lower. As a result, less compounds could be found in permeate and more in retentate. This gave wider range of substrates for anaerobic processing in UF retentates and faster organics conversion. In UF, much lower membrane resistance and shorter time of obtaining the assumed permeate recovery than in MF gave the evidence for fouling of mainly membrane surface, resulting from the proportion between pore size and diameters of retained particles. As a result, smaller load of solids was adsorbed in the membrane pores (Table 2) and bigger load remained in the retentate that was directed to anaerobic digestion. It was reported that dehydrogenase activity and specific methanogenic activity of biomass in an MBR was lower in the fouling layer and higher in the bulk sludge [47]. Also, microbial composition was different in these two sludges. In the present study, microbial activity, expressed as the ability of biogas production, differed in MF and UF retentate. In MF membrane, which was more susceptible to fouling than UF membrane, higher amounts of solids were retained in the pores and smaller amount remained in the retentate suspension. The transport of substrates in the dense fouling layer is suppressed and can lead to lower microbial activity [47]. This explained the differences in kinetics of biogas production from MF and UF retentates.

Disruption of particles may positively impact hydrolysis and accessibility of organic compounds, but on the other hand the composition of retentate is determined by the type of membrane and applied pressure, which may influence the biogas production. In the present study, anaerobic treatment of MF retentate gave 1.15–1.28 time lower cumulative biogas production and twice lower biogas production rate than the treatment of SS. Both in MF and UF, the period during which 90% of the cumulative biogas production was achieved increased to 13 days from 8 days obtained in the SS processing. This indicates that an increase in solubilization of organic matter cannot be directly related to an enhancement of biogas production, which was reported based on the study of sludge alkali-ultrasonication [48]. This was explained by the release of substances that impair the stability of anaerobic system, such as biorefractory components [49], excess ammonia [50], or some toxic compounds [51].

The rupture and lysis of microbial cells in activated sludge is protected by EPS and glycan strands crosslinked by peptides in cell walls. Disintegration of sewage sludge is considered to disrupt this stiff structure to accelerate hydrolysis of particles into dissolved molecules, thus liberating available nutrients and speeding up biogas production [52]. The operation of pumping devices in the MBR systems may be considered as such kind of pretreatment because sludge circulation through the membrane feed pump causes a significant decrease of the particle diameters. In the present study, the activated sludge flocs were estimated to have diameters mostly between 50 and 200 µm, whereas in retentates the flocs were totally dispersed, which made the size measurement impossible. This disruption of flocs was also visible by a substantial deterioration of retentate filtration through a paper filter. When sludge flocs are smaller, availability of substrates increases [53]. Therefore, the retentate obtained in filtration through ceramic membranes was expected to be a more effective substrate for anaerobic process than excess sludge from a secondary clarifier.

The lack of positive impact of sludge disintegration on kinetic parameters of biogas production indicates that degradation of flocs by high hydraulic shear force in membrane installation may decrease the biological activity of the sludge. This may result from the adverse effect of increasing mechanical strain on microbial symbiosis, particularly on the close relationship which is necessary for the interspecies transfer of hydrogen [54]. Disruption of juxta-positioning of the hydrogen producing bacteria and hydrogenotrophic methanogens enlarges the interspecies hydrogen transfer distance [24,55].

In practice, the susceptibility of SS separated by gravitational separation in secondary clarifiers to anaerobic conversion may differ from that separated by membranes because of different morphological sludge composition. Membranes effectively retain all bacteria, including filamentous ones. In the WWTP, which was the source of activated sludge for the present study, the abundance of *Curvibacter*-related filaments reached 15% of total biomass [21]. Biogas bubbles are trapped by the filaments bringing the microorganism to the digester surface and stabilizing the foam [56]. Strong binding between the filaments and gas bubbles deteriorates the release of biogas during digestion and negatively affects the operation of anaerobic system. Despite indisputably high efficiency of wastewater treatment in the MBRs, the feasibility of retentate digestion should also be taken into consideration when evaluating the performance of the whole system. 

In biogas produced from SS and MF/UF retentates, methane content was about 55%. The energy produced equaled 2.5–4.5 kWh/m^3^ feedstock from MF retentate and 4.4–4.8 kWh/m^3^ feedstock from UF retentate (Table 3). These values were lower by 4–50% than from the SS from the conventional excess sludge. Energy demand in MBRs varies widely because it depends on the configuration of the membrane module, flux level, packing density of membrane module, feed composition, MLSS concentration, membrane cleaning frequency, etc., [17,18]. Therefore, further studies in a well-controlled pilot-scale MBR would be necessary to evaluate if the energy generated in the present study satisfies the MBR energy demand.

## 4. Conclusions

The use of ceramic MF and UF membranes in place of a secondary clarifier resulted in obtaining permeates that could be suitable for reuse, for example in irrigation. High total efficiency of pollutant removal was neither influenced by the membrane *cut-off* nor by TMP. Despite smaller pore diameter, UF membrane was more hydraulically efficient. As a result of more intense fouling, MF membrane had 1.4–4.6 times higher filtration resistances than UF membrane. The membrane blocking decreased MF capacity but improved the permeate quality. Despite complete separation of activated sludge from the effluent, strong shearing forces in the membrane installation changed the kinetics of biogas production in comparison to the kinetics obtained when SS from a conventional system was anaerobically processed. Compared to the conventional activated sludge, MF retentates had 1.15–1.28 time lower cumulative biogas production. Anaerobic processing of MF and UF retentates resulted in about 60% elongation of period in which 90% of the cumulative biogas production was achieved. To develop MBR technology, long-term investigations to optimize biogas production from retentate are necessary.

## Figures and Tables

**Table 1:** Organization of the experiment.

Series	Type of Membrane	Pore Size (µm)/Membrane Cut-off (kDa)	TMP (MPa)
MF 0.2	MF	0.45 µm	0.2
MF 0.3	MF	0.45 µm	0.3
MF 0.4	MF	0.45 µm	0.4
UF 0.2	UF	150 kDa	0.2
UF 0.3	UF	150 kDa	0.3
UF 0.4	UF	150 kDa	0.4

**Table 2:** Hydraulic effects of membrane treatment.

Series	*J_V_* (L/(m^2^·h))	α (–)	*R_t_* (m^−1^)	*R_p_* (m^−1^)	*R_c_* (m^−1^)	Ads-TSS (%)	Ads-COD (%)
MF 0.2	15.0	0.005	4.8 × 10^13^	9.6 × 10^11^	4.7 × 10^13^	67.2	67.5
MF 0.3	44.3	0.009	2.4 × 10^13^	9.8 × 10^11^	2.3 × 10^13^	35.7	44.6
MF 0.4	55.7	0.009	2.6 × 10^13^	9.5 × 10^11^	2.5 × 10^13^	41.4	47.7
UF 0.2	68.9	0.070	1.0 × 10^13^	8.5 × 10^11^	8.9 × 10^12^	36.8	57.0
UF 0.3	72.9	0.050	1.5 × 10^13^	8.6 × 10^11^	1.3 × 10^13^	58.1	61.4
UF 0.4	76.4	0.040	1.9 × 10^13^	8.5 × 10^11^	1.7 × 10^13^	46.1	53.6

**Table 3:** Kinetic parameters of biogas production from sewage sludge (SS) and the retentates after MF and UF, and the energy produced from the feedstock.

Parameters		SS	MF 0.2	MF 0.3	MF 0.4	UF 0.2	UF 0.3	UF 0.4
Phase I	Cumulative biogas production after phase I (L/kg COD)	205 ± 6	160 ± 5	168 ± 7	178 ± 4	130 ± 4	124 ± 5	128 ± 6
	*k_I_* (d^−1^)	0.28 ± 0.01	0.16 ± 0.01	0.14 ± 0.01	0.13 ± 0.01	0.29 ± 0.01	0.46 ± 0.01	0.46 ± 0.01
	Duration of phase I (*t_I_*) (d)	21	21	21	21	9	8	7
	* *r_I_* (L/(kg COD⋅d))	57.1 ± 2.1	25.9 ± 3.2	23.5 ± 1.1	23.1 ± 0.9	37.7 ± 1.0	57.0 ± 2.4	58.9 ± 1.8
Phase II	Cumulative biogas production after phase II (L/kg COD)	–	–	–	–	208 ± 5	208 ± 6	214 ± 3
	*k_II_* (d^−1^)	–	–	–	–	0.22 ± 0.01	0.20 ± 0.01	0.17 ± 0.01
	Duration of phase II (*t_II_*) (d)	–	–	–	–	12	13	14
	* *r_II_* (L/(kg COD d))	–	–	–	–	17.2 ± 0.9	17.6 ± 1.1	14.6 ± 0.8
Energy yield (kWh/m^3^ feedstock)		5.0	2.5	4.5	4.5	4.4	4.5	4.8

* *r*—the initial rate of biogas production from 1st order model.
